# Microstructural, electrical and biological activity in $\mathrm{Ca}_{10}(\mathrm{PO}_4)_6(\mathrm{OH})_2-\mathrm{Ba}_{0.5}\mathrm{Sr}_{0.5}\mathrm{TiO}_3$ ceramic composites designed for tissue engineering applications

**DOI:** 10.1038/s41598-021-01748-8

**Published:** 2021-11-16

**Authors:** Apurba Das, Pamu Dobbidi, Aman Bhardwaj, Varun Saxena, Lalit M. Pandey

**Affiliations:** 1 Department of Physics, Indian Institute of Technology Guwahati, Guwahati, 781039 India; 2 Department of Physics, D K College, Mirza, Assam 781125 India; 3 Department of Biosciences and Bioengineering, Indian Institute of Technology Guwahati, Guwahati, 781039 India

**Keywords:** Implants, Biomedical materials, Ceramics, Composites

## Abstract

The article investigates electrically active ceramic composite of $\mathrm{Ca}_{10}(\mathrm{PO}_4)_6(\mathrm{OH})_2$ (HAP) and $\mathrm{Ba}_{0.5}\mathrm{Sr}_{0.5}\mathrm{TiO}_{3}$ (BST) for biomedical applications. The study is a systematic blend of the materials science aspect of composites with a special focus on the dielectric and biological properties and their relationships. The article emphasized primarily extracting the dielectric constant ($\epsilon _r)$ of the specimens (that lay in the range of 3–65) and related them to microstructural properties like the grain size and at.% of BST. A broad outlook on the importance of $\epsilon _r$ in determining the suitability of bioceramics for clinical applications is presented. Bioactivity analysis of the specimens led to probing the surface charges (that were negative), and it was found crucial to the growth of dense apatite layers. Furthermore, the cytocompatibility of the specimens displayed cell viability above 100% for Day 1, which increased substantially for Day 3. To reveal other biological properties of the composites, protein adsorption studies using bovine serum albumin (BSA) and fetal bovine serum (FBS) was carried out. Electrostatic interactions govern the adsorption, and the mathematical dependence on surface charges is linear. The protein adsorption is also linearly correlated with the $\epsilon _r$, intrinsic to the biomaterials. We delve deeper into protein–biomaterials interactions by considering the evolution of the secondary structure of BSA adsorbed into the specimens. Based on the investigations, 20 at.% HAP–80 at.% BST (20H–80B) was established as a suitable composite comprising the desired features of HAP and BST. Such explorations of electrical and biological properties are interesting for modulating the behavior of bioceramic composites. The results project the suitability of 20H–80B for designing electrically active smart scaffolds for the proposed biomedical applications and are expected to incite further clinical trials.

## Introduction

**Figure 1:**
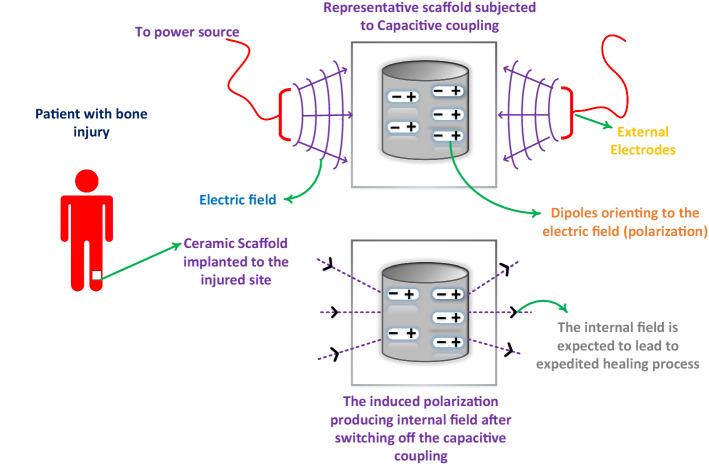
An illustration of the effect of ceramic scaffolds that inherently can undergo polarization when the external stimulations are applied upon the injured site for enhanced bone growth. The polarization helps maintain the electric field even after removing of the external field (capacitive coupling in this case). The extent of polarization directly depends upon the $\epsilon _r$ of the ceramics that can be tuned for achieving a stronger internal electric field. The possibility of such tuning can be done by adding another ceramic, which has been elaborated in the article. The illustration has been created using Visio 16 available at https://support.microsoft.com/en-us/office.

In the last few decades, an extensive impetus has been laid on developing biologically active materials for designing medical devices like scaffolds, electrets, and bone grafts. Electrical activity in biological systems such as piezoelectricity, streaming potentials, and ferroelectricity play a decisive role in regulating bone healing, repair, and regeneration through cell signaling^1,2^. The focus has recently shifted to the preparation of electrically active materials with superior biological properties that can augment the performances of the existing biomedical devices^3^. For instance, the electrically active biomaterials are explored for their capability in delivering drugs locally in a controlled and sustainable manner^4^. Recent interest in exploring electrical properties in biomaterials has also opened up the possibilities of their technological applications. In this regard, the recent discovery of piezoelectricity and ferroelectricity in hydroxyapatite $(\mathrm{Ca}_{10}(\mathrm{PO}_4)_6(\mathrm{OH})_2$, HAP) has led researchers to consider its future applications as electrets apart from bone tissue engineering^2,4^. Exploration of the electrical properties of HAP presents an exciting field of research. The electrical properties have also unfolded new ways for understanding the mechanism of bone formation, healing, and adaptive response of bones to an external stimulus like stress^5^. Specifically, the dielectric constant ($\epsilon _r)$ of an insulator plays a unique role in determining the role of biomaterials in clinical settings. An illustration of the idea is presented in Fig. 1.

The $\epsilon _r$ of HAP is typically on the lower side ($\simeq$5–15 at 1 MHz). It can be tuned by designing a ceramic composite of HAP with another ceramic having higher $\epsilon _r$. The bulk of the research in the ceramic composite is mostly focussed on mixing up a ferroelectric counterpart (of higher $\epsilon _r$) for improving the energy/charge storage capacity. However, such traditional ferroelectrics have a large value of remnant polarization that often decreases the amount of energy stored in these ceramics^6^. As the power consumption in implantable bioelectronics devices is usually low, therefore dielectrics with moderate permittivity ($\sim$30 to 100) could be useful for such implants^7^. $\mathrm{Ba}_{0.5}\mathrm{Sr}_{0.5}\mathrm{TiO}_{3}$ is an important component that can be considered for a composite with HAP. Composites of HAP with $\mathrm{BaTiO}_3$ have already been considered for biomedical applications^7^. The results are inspiring and have motivated us to consider the introduction of the Sr component in $\mathrm{BaTiO}_3$. Sr ions have a tremendous role in clinical applications as the use of drugs containing Sr nanoparticles have been useful in preventing vertebral, peripheral fractures and osteoporosis^8,9^. Recently, radio-pacifiers for implant fixation in the treatment of compression fractures have suggested the use of Ba as cross-linking agents^11^. The results are motivated by the fact that Ba as cross-linking agents have seen to significantly improve the radiopacity without any adverse effects on the cell viability of MG63 cells^10^. Therefore, including Ba and Sr in biological applications apart from HAP can be an interesting construct.

The biocompatibility in ceramics is regulated by a series of biointerfacial reactions like protein surface and cell-surface interactions. Various efforts are made to design multifunctional biomaterials (biomaterial composites) to modulate intermolecular interactions for tuning biointerfacial interactions^11^. Inspired by the developments, a ceramic composite of monoliths BST and HAP is synthesized by varying the concentrations of HAP and BST. By bridging the literature gaps, the study presented in this article is expected to provide new insights into the biological and physical processes in the presence of BST and HAP, simultaneously. However, considering the vastness of the subject matter, the article is focused only on exploring the electrical properties of ceramic composites and correlating them with their biological properties. To keep matters simple, the composites have been developed without adopting any high-end techniques for densifying the compacts^12^. Special priority on the dielectric properties has been given that are analyzed in the radio frequency (RF) regime: $10^6{-}10^9$ Hz. The motivation behind choosing the RF regime lies in its application in clinical settings for treating delayed unions and non-unions of bone fractures^13,14^. Understanding the dielectric properties in such a regime can provide vital inputs to clinicians for understanding the behavior of biomaterials before in-vivo applications. Furthermore, the protein adsorption on the surface of the composites is studied using bovine serum albumin (BSA) and fetal bovine serum (FBS) as the model proteins. The adsorption behavior is related to the dielectric properties, which is the unique aspect of the article. The ceramic–protein interactions are studied by understanding the evolution of the secondary structures of the adsorbed proteins. Such studies provide fresh perspectives in considering bioceramic composites with specific biological and electrical properties to design a new generation of advanced biomedical devices like electro-active scaffolds and self-healing bandages containing electrets.

## Experimental procedure

### Synthesis of the compact discs

The monoliths for preparing the composites were processed by two different methods. To prepare HAP, the sol–gel processing route was adopted using ethanol (Merck Laboratories, India) as the solvent for mixing the precursors $\mathrm{Ca}(\mathrm{NO}_{3})_{2}\cdot 4\mathrm{H}_{2}\mathrm{O}$ (Sigma Aldrich, USA) and $\mathrm{P}_{2} \mathrm{O}_{5}$ (Sigma Aldrich, USA) to maintain the molar ratio at 1.67. The second component, BST, was prepared following the conventionally adopted inexpensive solid-state processing route. The initial precursors required for the preparation consisted of $\mathrm{BaCO}_3$ (Sigma Aldrich, USA), $\mathrm{SrCO}_3$ (Sigma Aldrich, USA) and anatase $\mathrm{TiO}_2$ (Sigma Aldrich, USA), which were mixed in stoichiometric amounts according to the reaction

1 $$\begin{aligned} \mathrm{BaCO}_3+ \mathrm{SrCO}_3 + 2\mathrm{TiO}_2 \rightarrow 2\mathrm{Ba}_{0.5}\mathrm{Sr}_{0.5}\mathrm{TiO}_3 + 2\mathrm{CO}_2 \uparrow \end{aligned}$$

The details of the experimental procedure can be found in our earlier reports^15^. Once the precursors for the composites were ready, they were mixed in different at.% to prepare a series of compositions, for further characterization. For preparing the series, the mixing recipe adopted has been summarised in Table 1. For uniformly mixing the components, the precursors were weighed according to stoichiometry and mixed in a ball mill using the protocol described previously^15^. Poly-vinyl alcohol (PVA, Loba Chemie, India) was then added to the finely mixed powder serving as the prepared composites’ binding agent. The powders were then pressed into cylindrical green compacts in a hydraulic press (Technosearch instruments, India), applying a uniform pressure of 25 kg/cm$^{2}$. Several compacts were prepared for each composition under the same conditions and were finally sintered at 1073 K (3 h) for densification and compaction of the resultant cylindrical compacts.

**Table 1:** The composition of different green discs and their codes.

Monoliths		Scheme	Code
HAP	BST	100 at.% HAP	HAP
		20 at.% HAP & 80 at.% BST	20H–80B
		40 at.% HAP & 60 at.% BST	40H–60B
		60 at.% HAP & 40 at.% BST	60H–40B
		80 at.% HAP & 20 at.% BST	80H–20B
		100 at.% BST	BST

### Characterization of the surfaces

#### Micro structural, electrical and surface morphology

The characterization techniques adopted for a systematic analysis of the prepared specimens’ properties involved XRD for crystal structure evaluation. A diffractometer recorded the XRD patterns (Rigaku TTRAX III, Japan) by collecting the diffracted X-ray photons $(\lambda = 1.5406\, \AA )$. The diffracted X-ray photons were obtained for the specimens in the $(2\theta )$ range of $20^{\circ }{-}55^{\circ }$, scanning at the rate of $3^{\circ }$/min and collecting the data points at a frequency of $0.03^{\circ }$. The dielectric spectra for the prepared specimens were obtained at a frequency of 1 MHz, varying the temperature from 133 to 523 K using an impedance analyzer (Agilent Technologies 4991A, USA) equipped with a temperature control system (Novocontrol BDS 2300, Germany). The data points were collected at an interval of 10 K. The morphology of the specimens was observed using a FESEM (Sigma 300, Zeiss). The FESEM pattern were also used to determine the specimens’ grain sizes using the ImageJ software. The elemental composition of the specimens was also determined using the X-ray dispersive energy analysis (EDX, Oxford Instruments, UK) attached to the FESEM. To supplement FESEM images, the field emission transmission electron microscopy (FETEM, JEOL-JEM 2100F operated at 200 kV, Japan) images were recorded along with the high-resolution transmission electron microscopy (HRTEM) images.

**Figure 2:**
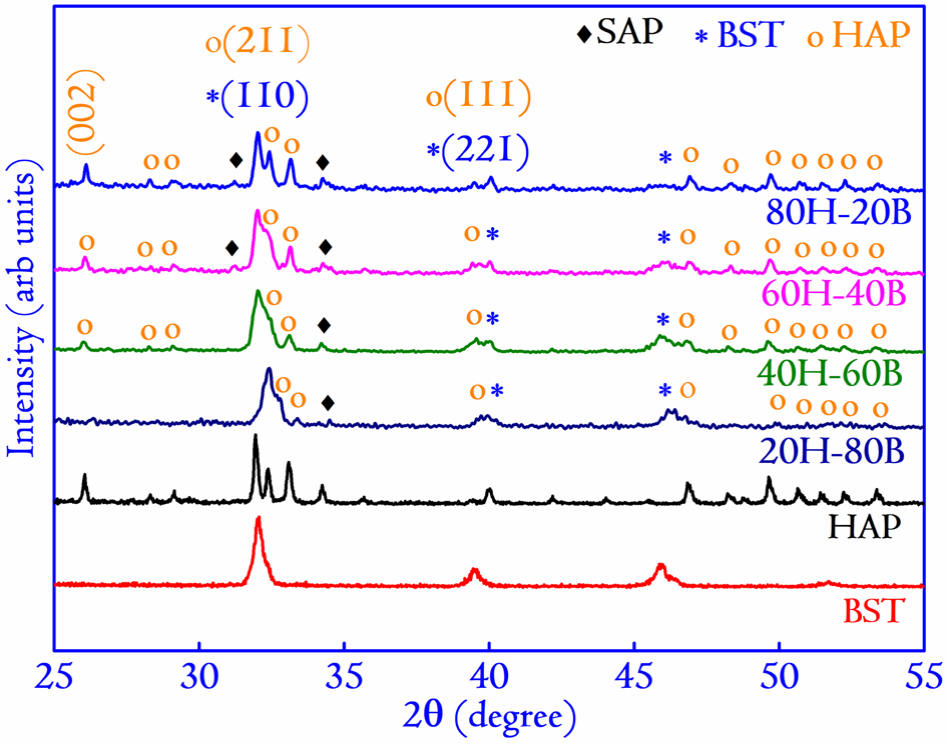
XRD patterns of the composites showing the peaks corresponding to both the monoliths along with the appearance of additional peaks corresponding to the SAP phase. The XRD patterns of the monoliths are also presented along with the composites. The patterns reveal a large extent of overlapping in the peaks due to the closeness of the Bragg’s angle in those positions. The dominance of the planes in the composite is observed, which corresponds to the more significant concentration of the monoliths. The (hkl) planes are identified by comparing the planes with the standard ICDD files: card no. # 39-1395 for BST, # 009-0432 for HAP, and card no # 002-0761 for SAP.

#### In vitro bioactivity and surface electricity

The in-vitro bioactivity of the specimens was analyzed using the simulated body fluid (SBF) solution. The specimens were incubated in 1.5 SBF solution for 12 days at 310 K, ensuring that the old solution was refreshed every 24 h. The 1.5 SBF solution was prepared using the protocol suggested by Kokubo et al.^16^. Following incubation, the specimens were washed and dried for 24 h at 333 K. Further, the growth of bone-like apatite was analyzed using FESEM for understanding the surface morphology of the apatite layer. The Zeta potential ($\zeta$) measurements were performed in a Litesizer particle size analyzer (Anton Paar, Austria) by dispersing the powdered form of the ceramics in deionized water at pH 7.4.

#### Protein adsorption, evolution of secondary structure and cytocompatibility analysis

Following the biomineralization process, the protein adsorption of the ceramic composites was analyzed. BSA, along with FBS, was used as the model protein for analyzing the protein adsorption on the surface of the synthesized discs as described elsewhere^17^. The procedure for studying the adsorption of proteins is discussed in brief. To start, BSA (500 $\upmu$g/mL) and FBS (10% v/v) solution were prepared in PBS. The specimens (compact discs) were weighed and incubated in protein solution for 1 h at room temperature for adsorption. The adsorbed protein was desorbed from the specimens by immersing them in 5% sodium dodecyl sulfate (SDS) solution by shaking incubation (180 rpm) at 310 K for another 1 h. The Bicinchoninic acid assay (BCA Assay kit, Sigma Aldrich, USA) was used to quantify the adsorbed proteins by measuring the absorbance at 562 nm using a microplate reader (Infinite 200 Pro, Tecan, Switzerland). The change in the secondary structure of the adsorbed proteins was further studied using the Fourier Transform Infrared (FTIR) analysis. The analysis was performed by recording the FTIR spectrum (Spectrum Two, Perkin Elmer, USA) in the Amide-I region (1590–1710 cm$^{-1}$). The observed spectra in the region were fitted with Gaussian curves to obtain the relative percentage of the secondary structures ($\alpha$-helix, $\beta$-sheet, $\beta$-turn, random coils, and side chains). The MTT assay on specimens at varying concentration (1.000, 0.500, 0.250 and 0.125 mg/mL), was carried out in sterilized MilliQ water on MG63 bone-like cell line in a 24 well plate, using the protocol previously described by our research group^18^. 1 $\times$ $10^{4}$ cells/well were seeded in each well and incubated for 24 h. Cells were incubated in Dulbecco’s Modified Eagle Medium (DMEM) supplemented with 10% FBS and 1% Penstrap antibiotic in a $\mathrm{CO}_2$ incubator at 5% $\mathrm{CO}_2$ and 310 K, after which the material was seeded into the wells. After each specified time interval, 20 $\upmu$L of MTT (0.5 mg/mL) was added to the wells following removal of the consumed DMEM media, and incubated in a $\mathrm{CO}_2$ incubator for 4 h. Post incubation, the formed formazan crystals were dissolved in 100 $\upmu$L filtered DMSO. Absorbance was measured at 570 nm (Infinite 200 Pro, Tecan). Cell viability (%) was calculated and compared to control wells (cells without any material incubation), taken as 100%.

## Results and discussion

### Structural analysis

XRD presents a non-destructive technique to probe the microstructure of synthesized composites. In the present study, the XRD patterns of the composite and monoliths have been shown in Fig. 2. The phase identification has been made by comparing the resultant XRD patterns with standard ICDD files (card no. # 39-1395 for BST and # 009-0432 for HAP)^15^. Apart from the monoliths, the peaks corresponding to $(\mathrm{Sr}_{5}(\mathrm{PO}_{4})_{3} (\mathrm{OH})$, SAP) (card no # 002-0761) are also seen, which presumably are formed due to substitution of Sr ions in the Ca site of HAP^15,19^. The intensity of peaks corresponding to SAP is highest for 80H–20B and decreases monotonously for the other composites in the series. The substitution might have occurred during the process of sintering at 1073 K. A possible reaction occurring between the monoliths that leads to the formation of SAP can be understood from the following equation

2 $$\begin{aligned} \delta \mathrm{Ca}_{5}(\mathrm{PO}_{4})_{3}(\mathrm{OH})+ 10 \mathrm{Ba}_{0.5}\mathrm{Sr}_{0.5}\mathrm{TiO}_3 \rightarrow 10 \mathrm{Ba}_{0.5\delta }\mathrm{Sr}_{0.5-0.5\delta }\mathrm{Ca}_{0.5\delta }\mathrm{TiO}_3 + \delta \mathrm{Sr}_{5}(\mathrm{PO}_{4})_{3}(\mathrm{OH}) \end{aligned}$$

However, it is interesting to note that the substitution of Ca by Sr is by no means complete, and the substitution occurs only for a small fraction of Ca. This substitution occurs due to the differences in the reduction potential of Ca (1.76 V) and Sr (1.95 V). Further, the standard reaction potential of Ba is higher than Ca, and the atomic radius of Ba (217 pm) is more significant than Sr (215 pm). Hence, it becomes difficult for the Ba ions to occupy a Ca site. Thus there is no substitution of Ca by Ba ions.

**Figure 3:**
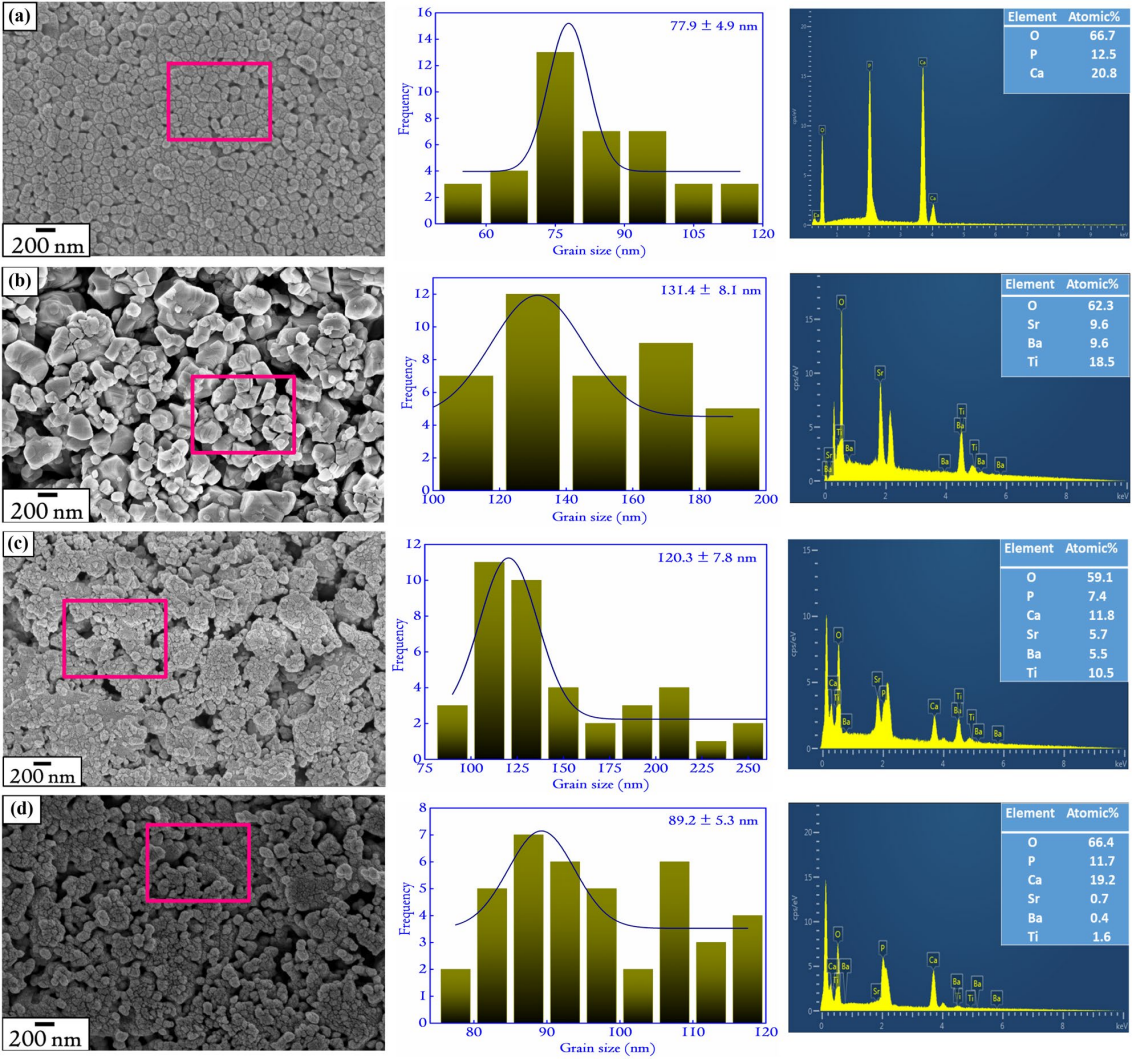
The FESEM patterns showing clearly the evolution of grains of the composites along with their monoliths. The variation in the grain sizes and EDX composition for (**a**) HAP, (**b**) BST, (**c**) 20H–80B, and (**d**) 80H–20B has also been included to get the quantitative estimate of the same. The grain size is the largest for the monolith BST. For the composites, the grain sizes lie in between BST and HAP. The Ca/P ratio in the specimens is 1.66, 1.64, and 1.59 for HAP, 80H–20B, and 20H–80B. The rectangles in the FESEM images shows the position from which the EDX signals have been acquired.

### Surface morphology and EDX analyses

The FESEM patterns of selected specimens are shown in Fig. 3. The patterns reveal that HAP is composed of spherical granulite with an average grain size of 77.9 ± 4.9 nm. The BST is composed of polyhedral structures with an average grain size of 131.4 ± 8.1 nm. The FESEM of BST contains pores that might have been incorporated due to the lower sintering temperature of 1073 K. Ceramics such as BST needs high sintering temperature with enhanced soaking time for achieving compaction, and a high-density^15,20,21^. However, in the present scenario, to avoid the generation of other phases of HAP (such as $\beta$-TCP), the sintering temperature is kept lower than 1273 K^15,18^. In the case of the composite, it is seen that the lower grain-sized HAP reduces the porosity present in between bigger-sized BST grains by occupying the voids. The grain sizes of the composites are expected to lie in between the monoliths HAP and BST^15^. The FESEM of the other composites have been included in the Supplementary File S1 in Fig. S1.

The EDX spectra of the composites and the monoliths are also shown in Fig. 3. The Ca/P values of the composites are calculated, and the Ca/P ratio shows a decreasing trend with the increasing concentration of BST in the composites. It is apparent that in case of composites with higher concentration of HAP, the EDX for Ca and P will be better leading to a higher Ca/P ratio in comparison to composites that have lower HAP concentration. The Ca/P ratio in HAP-based ceramics is vital in determining its biological properties in vivo^15^. A deviation in the Ca/P ratio from the value of 1.67 indicates variation in the dissolution behavior of the ceramics^15,22^. The dissolution behavior (rate of dissolution) is found to increase with the decrease in the Ca/P ratio of the ceramics^15,23^. For Ca/P ceramics, the ratio usually lies in between 1.50 and 2.00, and in this regard, the obtained values lie within the experimentally reported range^15,18^.

### Bioactivity and cell viability

**Figure 4:**
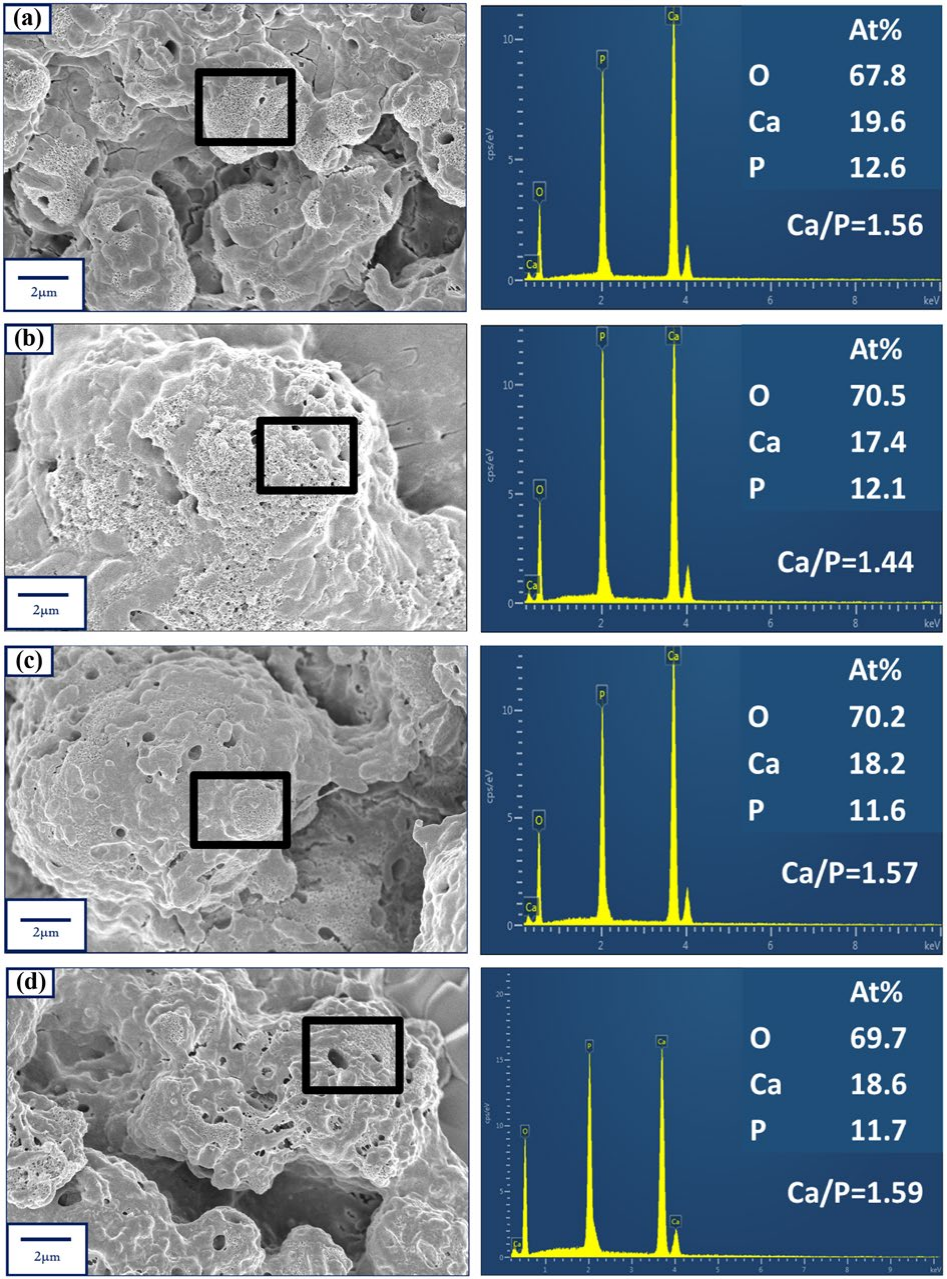
The FESEM micrographs showing clearly the nucleation of dense bone-like apatite structures along with micron-sized pores on the surface of (**a**) HAP, (**b**) BST, (**c**) 80H–20B and (**d**) 20H–80B. The corresponding EDX of the bone-like apatite is also shown which is highest for 20H–80B, implying that the synergistic effect of the Sr, Ba, Ti, $\mathrm{PO}_{4}^{3-}$ and Ca, enhances the biomineralization ability in the composite. The rectangles in (**a**), shows the position from which the EDX signals have been acquired.

The bioactivity of the specimens is determined by considering the ability of the discs to induce the growth of bone-like apatite on its surface when incubated in SBF^18^. The SBF tests are pretty successful in predicting the in-vivo performance of biomaterials as they can successfully create an environment that mimics the actual physiological conditions inside the host body^24^. The FESEM images of the bone-like apatite nucleated on the surfaces of the compact discs are shown in Fig. 4 for selected specimens. The FESEM images of the other specimens have been included in Supplementary File S1 (Fig. S2). In all the specimens, a dense growth of the apatite layer is observed from the micrographs. Naturally occurring bone apatite is characterized by a flowery structure with plate-like petals and numerous pores^24,25^. The apatite nucleated on the surfaces of the discs shows the mesoporous apatite layer formation with pore size in the range of microns. Such pores can help in the biointerfacial interactions of these structures when planted in-vivo as the pores are known to promote the ingrowth of bones^24,25^.

**Figure 5:**
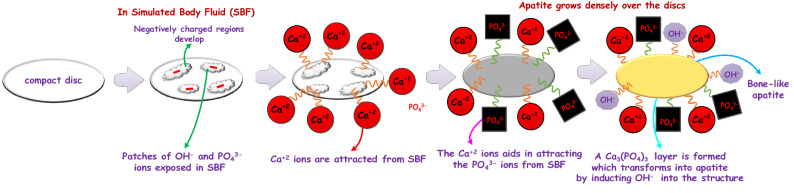
A schematic diagram of the probable mechanism of apatite nucleation in SBF over the surfaces of the discs. The nucleation mechanism is mainly related to the electrostatic forces that draw different ions from the SBF onto their surface. The illustration has been created using Visio 16 available at https://support.microsoft.com/en-us/office.

The mechanism of growth of apatite structures on compact discs can be understood by the chemistry and dynamics of the surface charges that build upon a surface when immersed in a fluid^26,27^. In most of the cases, it has been seen that the apatite nucleation starts with $\mathrm{Ca}^{2+}$ ions being nucleated on the surface resulting in the formation of Ca rich surface, which is positively charged^28^. This positively charged surface forms the base layer for drawing the negatively charged $\mathrm{PO}_{4}^{3-}$ ions by electrostatic interactions. Thus, an amorphous $\mathrm{Ca}{-}\mathrm{P}$ layer is generated, which has a high degree of solubility in water^18^. Therefore, from the point of thermodynamic stability, the amorphous $\mathrm{Ca}{-}\mathrm{P}$ layer would transform into a more stable bone-like apatite layer by incorporating $\mathrm{OH}^{-}$ ions from the surrounding SBF. Reports have earlier demonstrated that among the variants of $\mathrm{Ca}{-}\mathrm{P}$ ceramics, the apatite is the thermodynamically most stable form and its solubility in in-vivo or in-vitro surroundings is the least at physiological pH^18^. A graphical view of the probable mechanism of apatite growth is depicted in Fig. 5. Once nucleation of the apatite layer starts over the surface of the compact discs, a further increase in the apatite layer takes place by consuming the ions present in the SBF, which is supersaturated with respect to apatite crystals. It is to be understood that the most crucial step in the nucleation of the apatite layer on the surface of the discs is the drawing of $\mathrm{Ca}^{2+}$ ions from the SBF. The force of attraction must be electrostatic and as the $\mathrm{Ca}^{2+}$ ions are attracted towards the surface of the discs, it implies that the surfaces must be negatively charged. The $\zeta$ potential measurement is carried out to verify the proposition and the results reveal that the surfaces are indeed negatively charged. The numerically obtained values of $\zeta$ potentials for selected specimens are measured, and it is found to be − 21.97 mV, − 16.32 mV, − 35.64 mV, and − 13.42 mV for 20H–80B, 80H–20B, BST, and HAP, respectively. The negative $\zeta$ potential values confirm the probable mechanism stated earlier, by which apatite growth must have taken place over the surface of the discs. The negative $\zeta$ potential of the surfaces are in fact useful for inducing bone growth under in-vivo environments^29^.

In the case of the composites, the presence of Sr, Ba, Ti, and Ca are reported to delay the release of ions into SBF, which are later exchanged with $\mathrm{H}^{+}$ in the solution^29,30^. The slow dissolution leads to continuous apatite nucleation on the interface of the discs. In the composites containing $\mathrm{Ba}^{2+}$, $\mathrm{Sr}^{2+}$, $\mathrm{Ti}^{+4}$, and $\mathrm{Ca}^{2+}$, the biomineralization proceeds longer due to the additional $\mathrm{Ca}^{2+}$ ions. This reflects a higher $\mathrm{Ca/P}$ ratio of the composites compared to the monoliths^29,30^. In the case of the monoliths that contain lesser positive ions, reflected lesser Ca/P ratio. Amongst BST and HAP monoliths, the HAP surfaces offer thermodynamically favorable surfaces for apatite (Calcium Phosphate) formation due to similar nucleation centers that supports the growth of apatite layers^25^. This highly improves the Ca/P ratio of HAP compared to BST. The FESEM images in Fig. 4 shows that in the composites, the growth of the bone apatite layer is denser, which supports the proposition. In BST, the growth is scattered, and the plate-like structures are seen only in patches. The composites contain layers of apatite structures over which multiple other layers of apatite have been deposited. Such dense bone apatite growth in-vivo would be very much suitable for the integration of any biomedical devices designed from the composites. The Ca/P ratio of the deposited bone-apatite layer are highest for 20H–80B (1.59) and lowest for BST (1.44). Similar values of Ca/P ratio for bone-apatite are also reported by Sarma et al.^25^. The theoretical Ca/P ratio of HAP, an inorganic component of bone, is 1.67. The higher Ca/P ratio in the presence of composites (20H–80B and 80H–20B) reflected their bioactivity. The improvement in the Ca/P ratio of bone-apatite in composites containing HAP dictates the prominent role of surface charge and different ions in the nucleation of apatite from SBF, as shown in Fig. 5.

**Figure 6:**
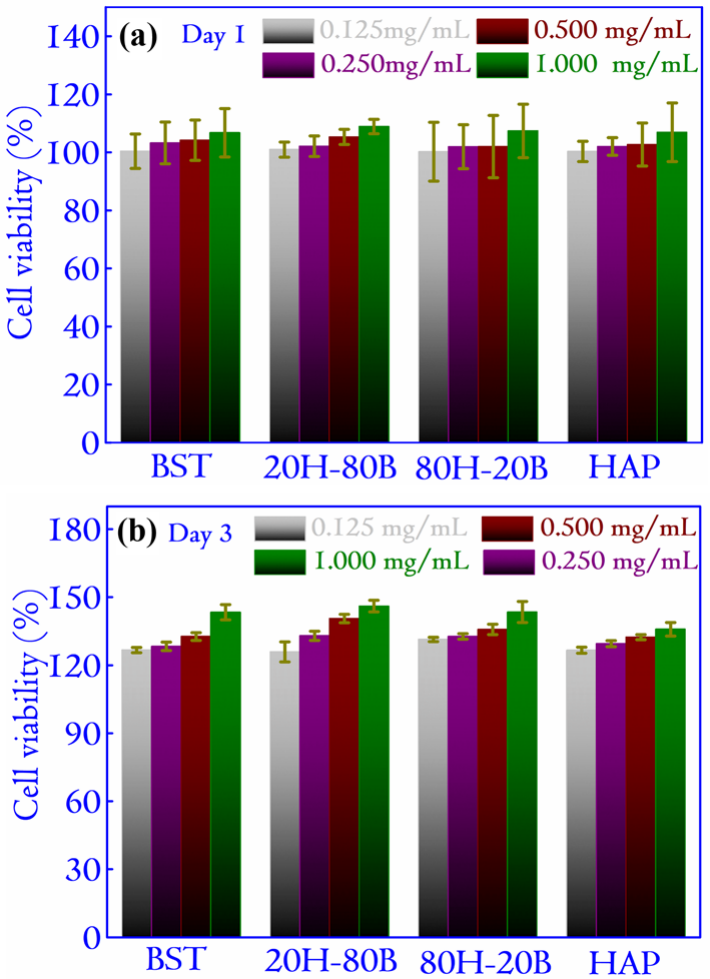
The cell viability of different specimens were studied with MG-63 cell lines for Day 1 and Day 3. All the specimens showed excellent viability amounting to values greater than 100% for all the concentrations. The higher cell viability of 20H–80B is mostly related to the presence of Ba and Sr ions, which have favored the proliferation of cells over the specimens.

The cytocompatibility analysis of the specimens is carried out using MTT assay on bone-like MG63 cell lines after incubating 1 $\times$ $10^4$ cells/well for 1 and 3 days. Varying concentrations of the specimens amounting to 0.125, 0.250, 0.500 and 1.000 mg/mL are considered and cell viability data are shown in Fig. 6. The specimens showed excellent cytocompatibility, and the cell viability was more than 100% compared to the control cells. Rather, the cell viability in the presence of material is higher than that of the control cells. In brief, the cell viabilities as compared to the controls are found to be 108.89, 107.39, 106.75, and 106.90% for 20H–80B, 80H–20B, BST, and HAP, respectively at 1 mg/mL for Day 1. The cell viability increases to 146.08, 143.47, 143.34 and 135.85% for 20H–80B, 80H–20B, BST, and HAP, respectively at 1.000 mg/mL for Day 3. These observations indicate the better cellular response in the presence of 20H–80B compared to other studied samples. Additionally, the cell viability is higher at a higher material concentration i.e. 1.000 mg/mL due to more concentration of ions. Comparatively high cell viability is observed on 20H–80B suggesting the presence of Ba and Sr have favored the growth of cells over such specimens^31^. It is observed that the presence of Sr and Ba along with HAP encourages the new bone formation and enhances cell viability^32^. In a similar study on BST-based scaffolds, it is established that BST has no negative effect on the viability of mammalian cells^20^. The presence of Sr is responsible for pre-osteoblastic cell differentiation, proliferation and also aids in suppressing osteoclastic differentiation. The overall high cell viability in the investigated ceramic composites and their monoliths demonstrates their cell proliferation ability and suitability in bone tissue regeneration applications.

### Dielectric properties

**Figure 7:**
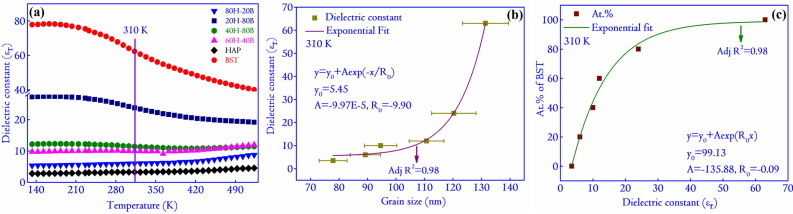
(**a**) The variation in the dielectric properties of the composites with respect to temperature at 1 MHz. The significant spike in the values is due to a probable phase transition of BST, and (**b**) shows the dependence of the $\epsilon _r$ (at 310 K) on the grain size of the composites. The relationship is found to be exponential with the adjusted R square value of 0.98. Similarly, (**c**), the variation of $\epsilon _r$ with respect to the at.% of BST in the composites is considered. The variation is again exponential, with the R square value of the fit at 0.98.

**Figure 8:**
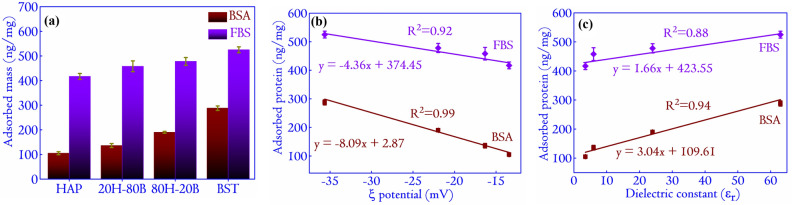
(**a**) The adsorbed mass of BSA and FBS on selected ceramic composites. The mathematical modeling of the behavior of the adsorbed proteins with respect to the $\zeta$ potential for (**b**) BSA and FBS follows a linear relation $y=mx+c$, with the parameters specified appropriately in the diagram. In (c), the variation of adsorbed proteins with $\epsilon _r$ is plotted. Similar to $\zeta$ potential, the variation with $\epsilon _r$ is also linear.

The variation in the dielectric properties as a function of the temperature of the composites and the monoliths are shown in Fig. 7a. The variation of dielectric properties versus temperature can reveal a plethora of information regarding the dipole dynamics that determine the electrical properties of ceramics^33^. For instance, the ceramic BST has a phase transition temperature of 230 K^34,35^. This causes an initial increase in the value of $\epsilon _r$ at a lower temperature, which gradually decreases with a temperature rise in the dielectric spectra (measured at 1 MHz) shown in Fig. 7a. This can be explained due to the phase transition of BST near 230 K, at which the $\epsilon _r$ shows a spike^35^. However, in the present scenario, the phase transition is incomplete and requires probing lower temperature for the transition to complete. The dielectric properties of ceramics are largely dependent on the physical properties such as grain size, and most importantly, the density^18,36,37^. To quantify the relationship between the $\epsilon _r$ and grain size, a plot between the same is shown in Fig. 7b. An exponential dependence is evident from the plot, and similar dependence of the $\epsilon _r$ with the at.% of BST is also considered, and it is observed that the dependence is exponential (shown in Fig. 7c). As no densification method has been adopted in this report, the density of the ceramics is expected to be very low (a large fraction of voids are observed in the FESEM micrographs of BST). Also, the temperature at which the ceramics are sintered is very low compared to the conventional ceramics that are fired above 1273 K for enhanced densification^38,39^. This might also reflect in the low value of $\epsilon _r$ in the case of BST, in contrast to the reports in the literature where BST is reported to have $\epsilon _r>$1000 at 310 K^40,41^. A similar value of $\epsilon _r$ ($\sim$ 70) is also reported for composites made of BST and poly(vinylidene fluoride-chlorotrifluoroethylene) when the BST concentration in the ceramic is varied from 0 to 50 vol.%^37^. Interestingly, with the increasing content of HAP, the transition is completely suppressed, and the value of $\epsilon _r$ remains constant for the entire temperature range probed in the experiment. The losses occurring in the composites and monoliths expressed by the term (tan $\delta$) at selected temperatures have been included in Table S1 in Supplementary File S1. The values of tan $\delta$ are observed to rise with temperature, and it may be attributed to the kinetic energy of the mobile charge carriers responsible for losses within the composites and monoliths.

The exciting correlation of dielectric properties with biological applications stems out from the recent advances in medical science, where the treatment of bone fractures has seen the application of an electric field as bone growth stimulators^42^. In orthopedics, the treatment of fractures, non-unions, and delayed unions are classic examples of complex and challenging scenarios for Clinicians and patients alike. The treatment of such complexities is accomplished by innovative techniques such as the applications of electrical fields^13^. The biology behind the healing mechanism of bone fractures in the presence of an electric field has not been understood (to a large extent) yet. Primarily, it is believed that the electric field applied at regular intervals accumulates ions such as $\mathrm{Ca}^{2+}, \mathrm{PO}_4^{3-}, \mathrm{OH}^{-}$ locally near the fractured region, which leads to the initiation and further progress of the highly critical bone-forming process^42^. However, case studies of patients subjected to electric fields have shown that the process is quite tedious. The electric field needs to be administered for a period of (at least) three months with an exposure time of 10 hours (minimum) daily^42^. The duration is undoubtedly lesser than the average healing time (without stimulations); however, extensive research is being carried out to decrease the healing time further. In this regard, it is proposed that the scaffolds designed from electrically active materials such as the one considered in this article can decrease the healing time. In bone tissue engineering applications, scaffolds are used for guiding bone growth and regeneration. When the electrical stimulation is used in conjunction with the scaffolds, the stimulation can trigger polarization. The polarized field can continue generating the electric field internally, even when the external field is removed. Scaffolds generated from ceramics are inherently capable of undergoing polarization and can sustain the polarized field for a sufficiently long time^13^. It can be expected that the continuous generation of an electric field in the injured sites will lead to accelerated recovery by the patients. The composites which show modest $\epsilon _r$ in the range of 3–65 can be potential candidates associated with an innovative modality for rapid fracture healing. However, apart from $\epsilon _r$, biocompatibility and bioactivity are crucial factors that need to be addressed. The idea is undoubtedly novel but requires a further in-depth analysis of several parameters related to the electric field and the electrical properties of biomaterials, which are currently under investigation by our research group.

### Protein adsorption and evolution of secondary structure

Protein adsorption is the first interaction at the interface of an implant in the host body. HAP is inherently adsorptive, and any implants into the host body that contains HAP would adsorb proteins from the physiological micro-environment^43^. These physiological proteins later support the binding of the cells^44^. The protein adsorption data on the monoliths and selected ceramic composites are shown in Fig. 8. The normalized concentration (by weight) of the adsorbed BSA is the highest on BST (amounting to 288 ± 9 ng/mg) and least on HAP specimens (amounting to 105 ± 6 ng/mg). The adsorbed FBS on the ceramic surfaces shows the same trend, with the highest value on BST amounting to 525 ± 12 ng/mg and least on the HAP specimens amounting to 417 ± 12 ng/mg. The more adsorbed amounts of FBS as compared to BSA on the individual specimens are due to higher initial protein concentration in case of FBS. The protein adsorption behavior on the ceramic surfaces is highly modulated by the surface properties such as the $\zeta$ potential. The $\zeta$ potential of the surfaces provides information about the surface charge properties in solution^45^. The Ca ions present in the HAP readily interacts with the BSA that has active Ca binding sites^46^, favoring the BSA–Ca interactions on the specimens interface. Furthermore, these interactions affect the protein structure and result in conformation changes upon adsorption^47^. Low conformational change leads to reversible protein adsorption and might be due to lesser protein adsorption. It causes less BSA to be adsorbed on the specimens containing Ca (usually depending on the relative (homogeneous) distribution of HAP in the composites). BST, on the other hand, consists of $\mathrm{Ba}^{2+}$, $\mathrm{Sr}^{2+}$ and $\mathrm{Ti}^{+4}$ positively charged ions. Thus the enhanced electrostatic interactions with negatively charged BSA molecules^48–50^ resulted in a higher adsorbed amount on BSA. The XRD analysis in section Structural analysis reveals the formation of SAP in the case of the composites. The presence of Sr in SAP is also responsible for the BSA adsorption. Since all the surfaces in this investigation have shown preferentially good amounts of adsorbed proteins (BSA and FBS), it is expected that these surfaces will induce faster bone-apatite growth on their surface, which is an essential condition for bonding the implants to the host site.

The mathematical dependence of the adsorbed protein with the electrostatic interaction properties of the ceramic surfaces (specifically the $\zeta$ potential) is important for understanding the adsorption dynamics. In the inception, the adsorption of a specific protein on a surface increases if some or all of the following criteria’s are fulfilled: (a) the concentration is very high, (b) the protein is structurally flexible, (c) quick diffusion due to low molecular weight and, (d) the repulsion between the surfaces and the incoming proteins is very low^51^. Figure 8b reflects a linear relationship between the $\zeta$ potential and the adsorbed mass of BSA and FBS. A linear increase in the BSA adsorption with a decrease in the $\zeta$ potential indicates that a negative $\zeta$ potential favors the adsorption of globular proteins. BSA is also a major component in the FBS ($\simeq$70 to 80%). Thus the adsorption of FBS followed the same trend as BSA adsorption^49,50^. As the electrical properties (specifically the $\epsilon _r$) is an intrinsic property related to the charge holding capacity of the material, the relationship between the adsorption and $\epsilon _r$ can be interesting to probe. The plots in Fig. 8b, c reflects a linear relationship as depicted by the $\zeta$ potential. Thus it can be concluded that the $\epsilon _r$ of a surface might positively correlate with its $\zeta$ potential and is also related to the protein adsorption behavior of the surfaces. Such investigations are rare and would be an interesting field of research to explore in future communications. We must note that protein adsorption in biomaterials is a complex scenario, and apart from $\zeta$ potential and $\epsilon _r$, factors like wettability and surface energy also have an important role^52,53^.

**Figure 9:**
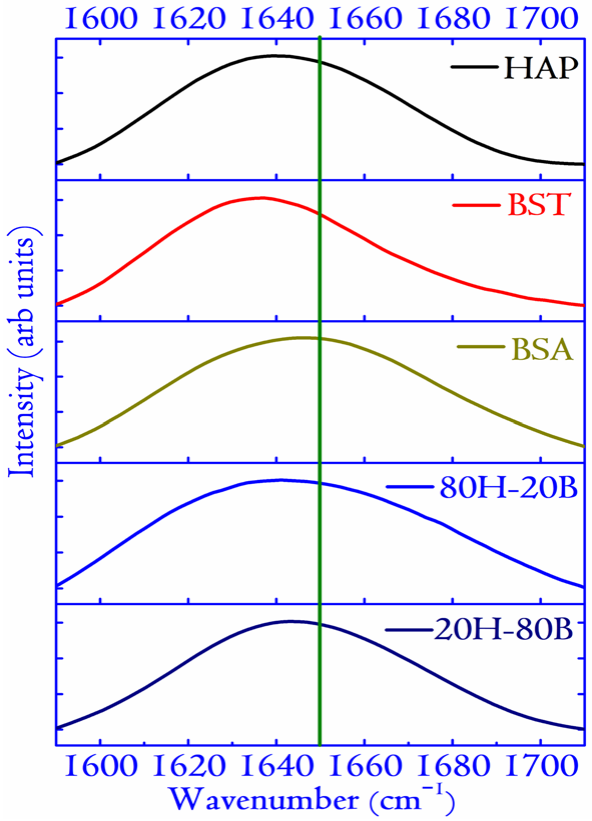
The FTIR spectra of BSA adsorbed surfaces in the Amide-I region (1580–1710 $\mathrm{cm}^{-1}$). The BSA concentration is fixed at 1.000 mg/mL for the adsorption experiments. The vertical line at 1650 $\mathrm{cm}^{-1}$ determines the shift in the maxima of the peak of BSA on adsorption to various surfaces. The shift is related to the conformational changes in the protein structure upon adsorption onto the surfaces of the compact discs.

**Figure 10:**
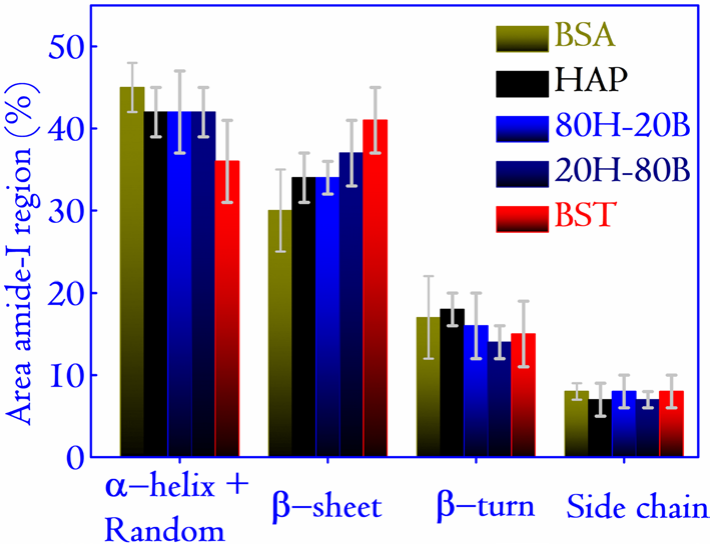
The variation in the content of secondary structures of BSA (in %) for various surfaces considered for protein adsorption. The content of secondary structurs are obtained by deconvoluting each of the FTIR spectra in Fig. 9 and the deconvoluted graphs are shown in Supplementary File S1 in Fig. S3.

For further in-depth analysis of the protein interactions with the synthesized ceramics, BSA is considered. The analysis of the secondary structure of the BSA reveals insight into adsorption behavior on the surfaces. Simultaneous with the surface adsorption of protein, conformational change and structural rearrangement of the protein take place. Hence, deconvolution of the Amide-I region as obtained from FTIR analysis is shown in Fig. 9. Native BSA contains primarily $\alpha$ helical structure along with random ($\sim$ 45%), $\beta$ sheet ($\sim$ 30%), $\beta$ turns ($\sim$ 17%), and side-chain ($\sim$ 8%)^17^. The range for the appearance of different secondary structures in the FTIR spectra of BSA are: $\alpha$-helix $\sim$ 1650 $\mathrm{cm}^{-1}$, $\beta$ sheet $\sim$ 1620–1636 $\mathrm{cm}^{-1}$, $\beta$ turns $\sim$ 1662–1688 $\mathrm{cm}^{-1}$ and a random peak is found $\sim$ 1645 $\mathrm{cm}^{-1}$ ^17^. The variations in the secondary structures for different surfaces are plotted in Fig. 10. Interestingly, it is observed that BST, which adsorbed the highest amount of BSA on its surface, displayed the least content of $\alpha$ helicity. The trend is evident in all the specimens, and HAP, which adsorbed the least amount of BSA, displayed the highest content of $\alpha$-helicity. Consequently, the content of $\beta$ sheet increased with the amount of adsorbed protein on its surface. The adsorbed protein on composite specimens resulted in higher helicity and lesser $\beta$ sheet contents than that on BST specimens. The secondary structures of the adsorbed protein on the composite specimens are comparable to those on HAP surface. However, the adsorbed amounts of proteins on the composite surfaces are enhanced up to 1.8 times compared to the HAP surface. These observations indicate better protein-surface interactions in the presence of composite specimens.

### TEM analysis

**Figure 11:**
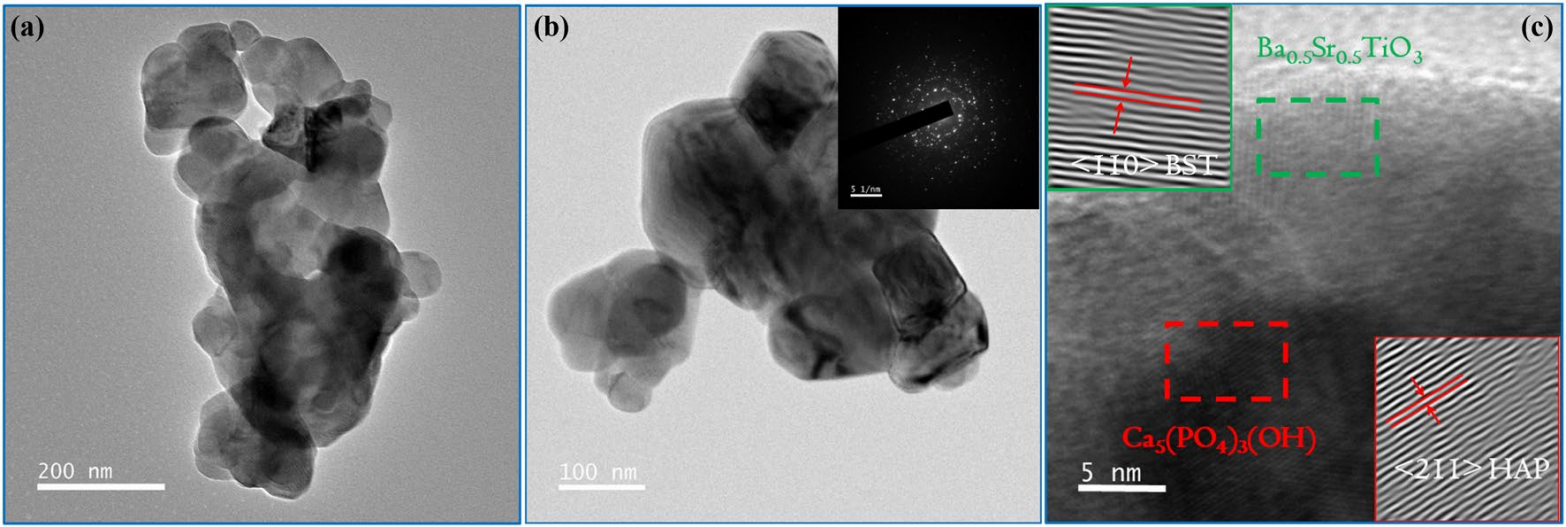
(**a**) The FETEM image of a selected region of 20H–80B showing the morphology of monoliths (**b**) the images of a different region at higher magnification as well as the SAED pattern of the region (inset) and (**c**) the HRTEM images of a different region along with their IFFT images in the inset. The lattice planes from the monoliths can be identified from the HRTEM images, as shown in (**c**).

**Figure 12:**
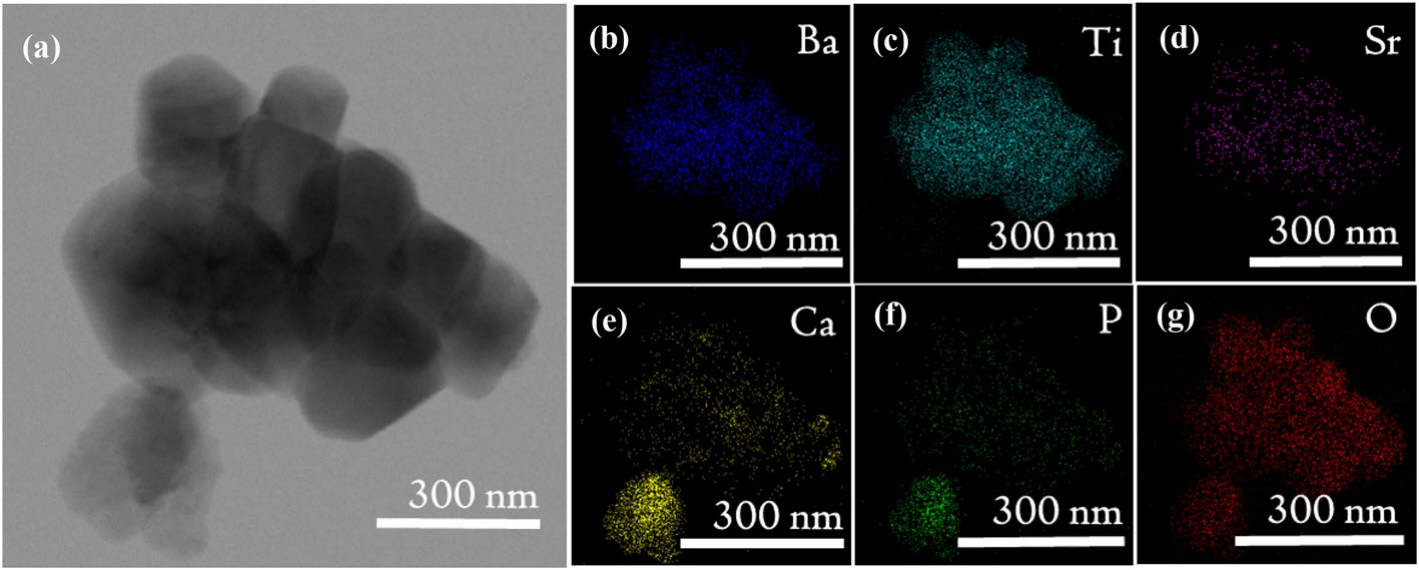
The STEM mapping of the region in Fig. 11b. The images show the distribution of different elements in the region. The distribution of the elements is shown in different colors, which helps to understand the presence of the elements in a different region of the STEM image. O being common to BST as well as HAP is distributed symmetrically.

Amongst the composites, 20H–80B exhibited the best dielectric properties. The $\epsilon _r$ of a composite directly demonstrates its ability to store electrical charges in it^6^. The higher value of $\epsilon _r$ usually implies a higher amount of charge storage capacity in the composite^6,34^. Such a biocompatible composite that has charge storage capacity can be highly useful in designing electrets for biological applications^54^. Electrets are highly used in biological experiments for electrical mediation in tissue growth and anti-thrombogenic surfaces^55^. Moreover, the dielectric properties of a material measure its ability to interact with electrical fields. Since the role of the electric field is instrumental in mediating the growth of bone cells, under in-vivo environments, electrically active scaffolds could play a prominent role^56^. The composite with higher $\epsilon _r$ also undergoes high degree of polarization on exposure to external electric stimulation, as discussed previously. Additionally, the growth of bone apatite over any prospective implants is necessary for osseointegration, and as shown in Fig. 4, the necessary apatite formation is obtained in the composites. However, the 20H–80B composite that blends the properties of HAP and BST shows the Ca/P ratio (1.59) of the bone apatite closest to the stoichiometric apatite (1.67). A higher degree of bioactivity is beneficial for our study as it would directly imply the superior bonding ability of the material with the tissue, thereby increasing the interfacial strength^32^. Based on the dielectric properties and bioactivity, the composite 20H–80B is endowed with superior biological and physical properties and considered for further investigations. The choice of 20H–80B is also supported by the cell viability (highest for 20H–80B) and protein adsorption studies that display significantly high values for the specimen. Accordingly, to gain a better understanding of the crystal structure, the FETEM analysis is carried out. In Fig. 11a, b, the images in two specific areas at two different magnifications have been shown along with the selected area electron diffraction (SAED) pattern of the composite in Fig. 11b. In Fig. 11c, the HRTEM images of the composite are displayed, and lattice fringes corresponding to both the precursors are seen that corresponds to (110) plane of BST and (211) plane of HAP. The lattice fringes are generated by inverse fast Fourier transform (IFFT) of the regions indicated by the dotted rectangle, and the corresponding inter-planar spacing has been compared to the standard ICDD files of HAP and BST to identify the corresponding (hkl) planes. The planes also appear in the XRD analysis shown in Fig. 1 and thus complements the XRD analysis. The high-resolution surface features of the composite are studied by scanning transmission electron microscope (STEM) mapping, which gives the distribution of various elements and has been included in Fig. 12a–g. The area selected for mapping analysis is chosen from Fig. 11b that contained BST and HAP.

In Fig. 12a–g, larger grains that are visible in the STEM image correspond to the BST grains. The HAP grains are distributed evenly that can be confirmed from the distribution of Ca and P (Fig. 12e, f). However, the presence of HAP is more prominent in the lower part, where the distribution is denser. The distribution proves that the HAP agglomerates in this region, which are quite expected as the grain sizes of HAP are in the nano-domain. The surface-to-volume ratio of the nanoparticles is usually large, leading to an increased level of surface energy. So, by agglomeration, an interface with lower energy is formed to reduce the surface energy, thereby stabilizing its present form.

### Conclusions

In summary, a series of composites with varying concentrations of BST and HAP is synthesized successfully, and the composite with enhanced biophysical property is identified. Microstructural analysis of the composites and monoliths using XRD reveals details of the composite structures. An interesting observation is apparent: the formation of SAP, in the composites due to the substitution of a small fraction of Ca by Sr. The FESEM reveals numerous pores in the synthesized composites and monoliths, and the grain size derived from it are found to increase with the concentration of BST. All the specimens could induce bone apatite from SBF, which is desirable for biomedical applications. The capacity to nucleate bone apatite from SBF directly points at a high degree of bioactivity. The presence of more cationic and anionic sites in the composites favored biomineralization of bone apatite compared to pristine BST and HAP. This resulted in dense apatite growth in the composites compared to the monoliths. The dielectric spectra of the specimens show the $\epsilon _r$ to lie in the range of 3–65 and low density of the specimens was identified as the significant factor in lowering the $\epsilon _r$. The modest values of $\epsilon _r$ can be harnessed to generate a polarized electric field, contributing to the expedited healing process of bone injury. Protein adsorption studies on the specimens show the highest concentration of BSA adsorption on BST. The surface charges ($\zeta$ potential) on the ceramics dictate the composites’ adsorption behavior, revealing a linear dependence (versus adsorbed mass for BSA). FBS adsorption showed a similar trend to BSA and is attributed to the major component (BSA) in FBS. The variation in the content of the secondary structures of BSA adsorbed on the ceramic surfaces is studied by the FTIR. The deconvolution results pointed at the presence of $\beta$-sheets, $\alpha$-helix, and $\beta$-turn in line with several studies conducted earlier. Based on the protein adsorption studies, we conclude that under in-vivo environments, the composite properties will be modulated by the collective behavior of the two monoliths. The exploration of the relationship between adsorbed proteins with $\epsilon _r$ and $\zeta$ potential generated a linear mathematical model into the complex interactions of proteins with biomaterials surfaces. Furthermore, the MTT studies revealed that specimens under investigation had cell viability above 100%, a factor crucial for the cell proliferation ability of the composites. In this context, Ba, Sr, Ti alongside HAP is also instrumental in new bone formation and enhancing cellular proliferation. Inspired by the results presented, we conclude the suitability of 20H–80B for designing bioelectrets and electrically active scaffolds.

## Supplementary Information

Supplementary Information.

## Data Availability

The datasets generated during and/or analysed during the current study are not publicly available due to the ongoing research related to the materials under investigation but are available from the corresponding author on reasonable request.
