# Supplementary material for: Microstructural, electrical and biological activity in Ca10(PO4)6(OH)2 - Ba0.5Sr0.5TiO3 ceramic composites designed for tissue engineering applications
Source: Sci Rep. 2021 Nov 16;11:22304. doi: 10.1038/s41598-021-01748-8 (PMC8595382; doi:10.1038/s41598-021-01748-8)
Supplement: Supplementary file 1 — Supplementary Information. [file 41598_2021_1748_MOESM1_ESM.pdf]

Supplementary File S1

Microstructural, electrical and biological activity in $Ca_{10}(PO_4)_6(OH)_2$ - $Ba_{0.5}Sr_{0.5}TiO_3$ ceramic composites designed for tissue engineering applications

Apurba Das[1,2], Pamu Dobbidi[1,*], Aman Bhardwaj[3], Varun Saxena[3], and Lalit M Pandey[3]

[1]Department of Physics, Indian Institute of Technology Guwahati, Guwahati-781039

[2]Department of Physics, D K College, Mirza-781125, Assam

[3]Department of Biosciences and Bioengineering, Indian Institute of Technology Guwahati, Guwahati-781039

[*]pamu@iitg.ac.in

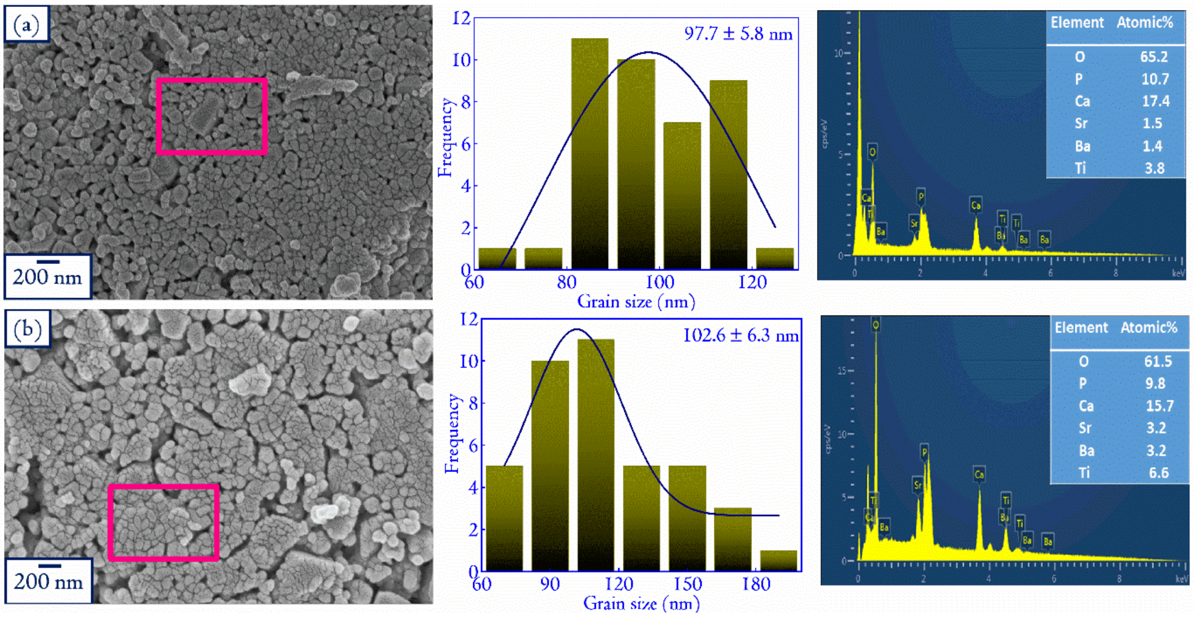

Figure S1: FESEM micrographs of (a) 60H − 40B and (b) 40H − 60B along with their respective EDX spectrum. The EDX has been done in the area shown by the rectangle.

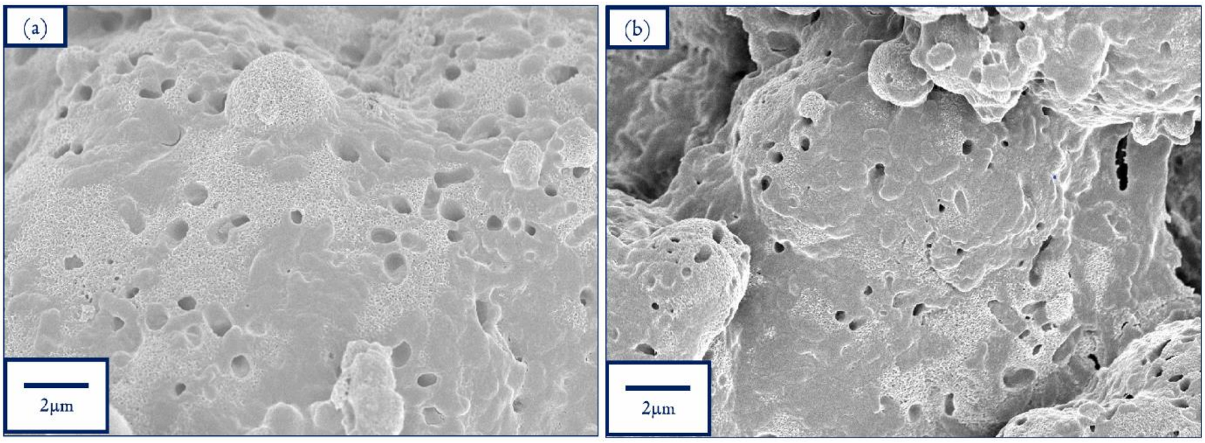

Figure S2: FESEM micrographs of (a) 40H − 60B and (b) 60H − 40B composites incubated in SBF.

Table S1: The variation in the dielectric loss factor (tan δ) at selected temperatures at 1MHz.

	Temperature (K)	***283***	***323***	***373***	***423***	***473***
Specimens	***BST***	0.36	0.45	0.41	0.41	0.46
	HAP	0.20	0.24	0.27	0.63	1.03
	$\mathbf{20}\boldsymbol{H}-\mathbf{80}\boldsymbol{B}$	0.34	0.37	0.42	0.61	0.79
	$\mathbf{40}\boldsymbol{H}-\mathbf{60}\boldsymbol{B}$	0.25	0.21	0.27	0.28	0.35
	$\mathbf{60}\boldsymbol{H}-\mathbf{40}\boldsymbol{B}$	0.26	0.41	0.66	0.74	1.13
	$\mathbf{80}\boldsymbol{H}-\mathbf{20}\boldsymbol{B}$	0.21	0.22	0.24	0.45	0.57

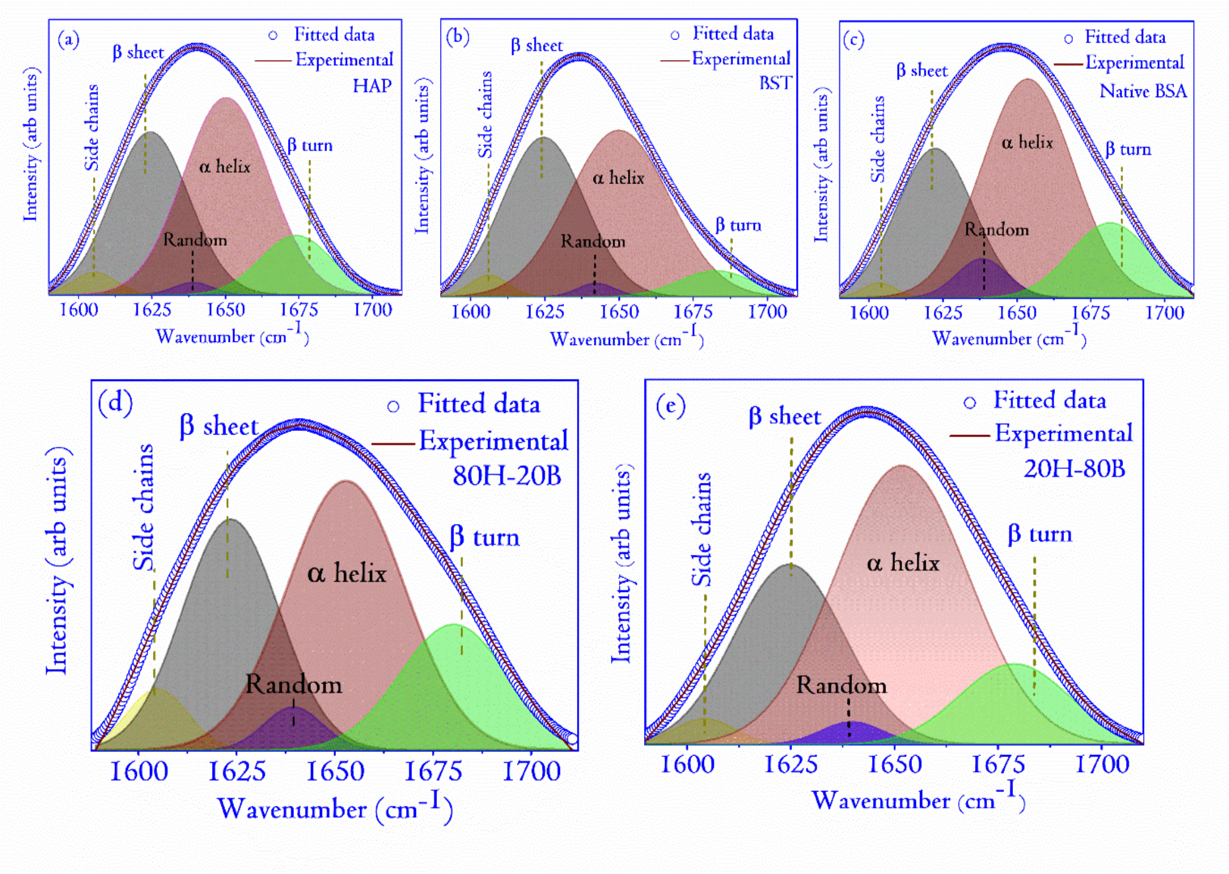

Figure S3: The deconvolution of the FTIR spectra to quantify the variation of the secondary structures of BSA in the considered specimens.
